# Reaction on “Ocular ultrasound versus MRI in the detection of extrascleral extension in a patient with choroidal melanoma”

**DOI:** 10.1186/s12886-019-1206-y

**Published:** 2019-08-27

**Authors:** M. G. Jaarsma-Coes, T. A. Ferreira, G. P. M. Luyten, J. W. M. Beenakker

**Affiliations:** 1 Ophthalmology, Leiden University Medical Center, P.O. 9600, 2300 RC Leiden, The Netherlands; 2 Radiology, C.J. Gorter Center for High Field MRI, Leiden University Medical Center, P.O. 9600, 2300 RC Leiden, The Netherlands

**Keywords:** MRI, Uveal melanoma, Extrascleral extension, Ultrasound

## Abstract

**Background:**

In the recently published article entitled “Ocular ultrasound versus MRI in the detection of extrascleral extension in a patient with choroidal melanoma” Jacobsen et al. describe a case in which a hyper-intense extra-ocular lesion on MRI was erroneously diagnosed as an extrascleral extension of the tumor. Based upon this the authors conclude “the superiority of ocular ultrasound in the diagnostic management of extra scleral extension in choroidal melanoma”. In our view, there are numerous flaws in the investigation that cast doubt on this message.

**Main:**

First of all, this is quite a bold statement when only one patient has been evaluated. Secondly, the manuscript only presents a post-contrast T1-weighted image, whereas multiple MRI-sequences need to be included to determine if a hyperintense region is an extrascleral invasion. Moreover, no modern MRI-techniques such Dynamic Contrast Enhanced (DCE) or Diffusion Weighted Imaging (DWI) have been included in the evaluation of this patient, making it hard to use this single case to compare the efficacy of MRI and Ultrasound. The presented data do, however, give clear clues that the hyperintense lesion is likely to be inflammatory.

**Conclusion:**

Although the study falls short in providing a comprehensive comparison between current MRI techniques and ultrasound, it does show that the evaluation of ocular MR-images should be made in a multi-disciplinary setting involving both ophthalmologist and radiologists, since the field of ocular MRI is continuously progressing.

## Background

Given the recent attention on the use of MRI for the diagnosis of ocular lesions, especially for uveal melanoma, the article of Jacobsen et al. [1] is most timely. It is furthermore commendable of the authors to share their experience of an erroneous diagnosis, as, in general, this could prevent such a misdiagnosis in the future. Nevertheless, in our view, there are numerous flaws in the investigation that cast doubt on its main message “to describe the superiority of ocular ultrasound in the diagnostic management of extra scleral extension in choroidal melanoma”, besides the fact that this is quite a bold statement when only one patient has been evaluated.

## Main text

In the presented post-contrast MR-Image indeed an abnormal hyperintense lesion can be seen at the arrow, but to conclude that this is an extrascleral invasion, multiple other sequences need to be included in the evaluation. First the pre-contrast T1 needs to be included to verify that this lesion is indeed enhancing, as it could already be hyperintense before contrast, similarly to intra-ocular hemorrhage possibly present in the temporal half of the vitreous. Secondly a T2-weighted image should be included, as UM is generally hypointense, while the signal intensity of inflammatory lesions depends on the balance between oedema and fibrosis, which can be used to differentiate between different types of orbital inflammation [2]. Although these basic MR-contrasts, and more advanced sequences such as diffusion weighted imaging (DWI), have not been included, the presented post-contrast T1-weighted image gives two clear clues that the hyperintense lesion is not an extra-scleral extension of the UM. Firstly, the lesion is located quite temporally from the tumor, making it less likely to be extrascleral invasion. More importantly, however, the sclera can be appreciated as a continuous hypointense line around the complete eye, excluding the possibility of scleral invasion of the tumor, since this would result in a hyperintense region in the sclera, connecting the main tumor and the extrascleral extension.

Moreover, we have some concerns about the correlation between the MR-image and the presented ultrasound and histology images. The ultrasound and histology, only show a small part of the tumor and eye and no guarantee can be given that they include the region with the hyperintense lesion, especially since they could have been acquired perpendicularly to axial MR-slice.

In our opinion the lesion would more likely be an artifact or inflammatory. However, we have never seen a type of artifact like this before in literature or in our clinic so inflammation is more likely. It could be periscleral cellulitis caused by sclerites which is more likely than an artifact as the patient also complains about pain. Unfortunately, no modern MRI-techniques such Dynamic Contrast Enhanced (DCE) or DWI have been included in the evaluation of this patient. DWI is one of the main diagnostic tools to differentiate between benign and malignant enhancing lesions and is valuable for diagnosing inflammatory disease [2], while other quantitative MRI-techniques, such as the commonly used DCE imaging, have been shown to differentiate between different orbital lesions [3].

Although MRI has been proposed to evaluate UM from the eighties [4, 5], in the last decade significant technological advances have been made, which have significantly improved the quality and diagnostic performance of ocular MRI [2, 6–9]. Unfortunately, in the discussion the most-recent cited MR-related article is more than 15 years old. Based on modern literature and our experience it is recommended to use local receive coils for the evaluation of small anatomies, such as the eye, since they yield an increased image quality compared to the head coil which was used in this study [9]. For ocular imaging a local receive coil, which allows for a smaller field-of-view, has an additional benefit as it reduces the imaging time, resulting in less eye-motion artefacts. Using these advances in ocular MRI, images with an resolution of < 0.5 × 0.5mm^1^ can easily be acquired at clinical 3 Tesla MRI, while at 7 Tesla an additional four-fold increase in resolution can be achieved [9].

## Conclusions

Although the study falls short in providing a comprehensive comparison between current MRI techniques and ultrasound, it does show two points that require more careful attention. Firstly, since the field of MRI in general, and ocular MRI specifically, is progressing continuously, the evaluation of ocular MR-images should be made in a multi-disciplinary setting involving both ophthalmologist and radiologists. Secondly, new studies are needed to assess if the older conclusion on the clinical value of MRI for uveal melanoma are still valid, as the diagnostic capabilities of MRI have significantly increased over the last years.

## Data Availability

Data sharing is not applicable to this article as no datasets were generated or analysed during the current study.

## Abbreviations

DCE: Dynamic contrast enhancement
DWI: Diffusion weighted imaging
MRI: Magnetic resonance imaging
T: Tesla
UM: Uveal melanoma
